# Heparin-binding protein and Endothelin-1 in critical COVID-19

**DOI:** 10.1186/s12871-026-04050-0

**Published:** 2026-06-23

**Authors:** Halla Halldorsdottir, Jesper Eriksson, Olav Rooyackers, Jonathan Grip, Johan Mårtensson, Eddie Weitzberg, Anders Oldner

**Affiliations:** 1 Department of Physiology and Pharmacology, Section of Anaesthesiology and Intensive Care, Karolinska Institutet, Stockholm, Sweden; 2 Department of Clinical Sciences, Division of Anaesthesia and Intensive Care, Karolinska Institutet, Danderyd Hospital, Danderyd, Sweden; 3 Department of Clinical Science, Intervention and Technology (CLINTEC), Division of Anaesthesia and Intensive Care, Karolinska Institutet, Stockholm, Sweden; 4 Department of Perioperative Medicine and Intensive Care, Karolinska University Hospital, Stockholm, Sweden

**Keywords:** Heparin-binding protein, Endothelin-1, COVID-19, Invasive mechanical ventilation, Intensive care unit, Mortality

## Abstract

**Background:**

Heparin-binding protein (HBP) is an inflammatory protein released by activated polymorphonuclear white cells. It has been suggested as a predictor of sepsis progression and organ dysfunction and plays a role in the pathophysiology of endothelial dysfunction. Endothelin-1 (ET-1) is a potent endothelium-derived vasoconstrictor with pro-inflammatory effects, and high levels are found in patients with sepsis and acute respiratory distress syndrome. We investigated HBP and ET-1 plasma levels in critical COVID-19 disease with the aim of evaluating whether they were associated with 60-day mortality or the need for invasive mechanical ventilation (IMV). These levels were compared with those of a cohort of post-trauma intensive care unit (ICU) patients.

**Methods:**

We included 96 patients with critical COVID-19 disease in 2020 and ten post-trauma ICU patients. Blood samples were collected at ICU admission, and plasma levels of HBP and ET-1 were measured. Clinical and laboratory data were collected until ICU discharge or death.

**Results:**

In COVID-19 patients, plasma levels of HBP were markedly increased, with a median level of 150 ng/ml (IQR 47–299), compared to 13.3 ng/ml (IQR 8.8–62.1), *p* < 0.0001 in the trauma ICU patients. There was no association between HBP levels and 60-day mortality or need for IMV. The levels of ET-1 were 1.6 pg/ml (IQR 1.2–1.9) in the COVID-19 cohort and 2.0 pg/ml (IQR 1.2–2.8), *p* = 0.25 in the trauma ICU cohort. COVID-19 patients requiring IMV hade higher ET-1 levels than those who did not require such treatment; however, no association was found in a logistic regression model when adjusted for age, sex and body mass index. There was no correlation between plasma HBP and ET-1 levels. Inflammatory parameters such as C-reactive protein, procalcitonin, ferritin, and interleukin-6, were elevated but did not distinguish survivors from non-survivors.

**Conclusion:**

While HBP levels are markedly elevated in critical COVID-19, they do not predict outcomes at ICU admission. ET-1 levels were also not linked to mortality or the need for IMV.

**Supplementary Information:**

The online version contains supplementary material available at 10.1186/s12871-026-04050-0.

## Introduction

The COVID-19 pandemic has posed significant challenges to healthcare systems worldwide. While the pathophysiology of COVID-19 is still being elucidated, numerous studies have documented that patients who initially appear relatively stable can experience rapid clinical deterioration within a few days. This decline is often characterised by uncontrolled pulmonary inflammation, acute respiratory distress syndrome (ARDS), and multiple organ failure [1–4].

The concept of a cytokine storm, marked by elevated levels of cytokines, has been proposed as a contributing factor to this process [5, 6]. However, virus-mediated damage, combined with dysregulated inflammatory responses involving non-cytokine biomarkers, is also recognised as playing a significant role in disease progression [7–10].

The ARDS associated with COVID-19 involves a complex interplay of epithelial and endothelial cell injury, leading to fluid leakage into the interstitium and alveoli [11, 12]. This vascular damage precipitates systemic endotheliitis, leukocyte adhesion, platelet activation [12–14], and the development of a pro-thrombotic state [14–16]. The specific contributions and temporal dynamics of epithelial versus endothelial injury in COVID-19 remain unclear, potentially causing variations in circulating plasma markers throughout the disease course [17].

The neutrophil protein heparin-binding protein (HBP) has emerged as a biomarker in sepsis, demonstrating the ability to predict unfavourable outcomes such as multiple organ failure and mortality [18–22]. Plasma concentrations > 15 ng/ml at intensive care unit (ICU) admission have been associated with increased mortality [22]. Moreover, plasma HBP levels are significantly elevated in viral influenza A (H1N1) pneumonia [23].

In the context of COVID-19, HBP elevation has been observed before the onset of organ failure when sampled early in the disease course [24]. Furthermore, elevated HBP levels have been linked to severe respiratory failure and increased mortality rates [24–26].

Heparin-binding protein is stored in secretory and primary granules in polymorphonuclear neutrophils (PMN). This multifunctional inflammatory protein is released when PMNs are activated through membrane bound β-2 integrin binding on the endothelial lining [27] or by circulating proteins such as streptococcal M-protein and fibrinogen [28]. Heparin-binding protein induces cytoskeletal changes in the endothelial lining, leading to intercellular gap formation, increased vascular permeability, and organ failure [27, 29].

Dysregulation of the endothelium-derived vasodilator nitric oxide (NO) and the vasoconstrictor peptide endothelin-1 (ET-1) [30] has been implicated in the pathophysiology of COVID-19-associated endothelial dysfunction, resulting from the direct invasion of endothelial cells by the virus [14, 31, 32].

Endothelin-1 serves as a potent inflammatory vasoconstrictor mediator released primarily by endothelial cells [33]. Elevated ET-1 levels have been correlated with conditions such as hypertension [34], heart failure [35], pulmonary hypertension [36, 37] and sepsis [38]. In pulmonary arterial hypertension, increased ET-1 levels are associated with fibrosis and vascular remodelling, suggesting a potential benefit of ET-1 receptor antagonists [39].

Numerous studies have demonstrated elevated ET-1 levels in ARDS [40–42]. Recent evidence suggests that patients with mild COVID-19 infection have lower ET-1 levels compared to hospitalised counterparts [30]. However, ET-1 levels can remain persistently elevated up to three months post-COVID-19, coinciding with increased inflammatory cytokine levels [43]. Higher ET-1 levels have been linked to severe COVID-19 and mortality [30, 43–45].

Supporting a pathophysiological role of ET-1 in COVID-19, a recently published study showed that the dual ET_A_ and ET_B_ receptor antagonist bosentan reduced the risk of hospitalisation and thrombosis in high risk COVID-19 patients [46].

Despite data suggesting the potential involvement of ET-1 and HBP in the pathophysiology of critical COVID-19, their role remains largely unclear [47], with a limited number of studies assessing the levels of these biomarkers in COVID-19 patients. In particular, data on biomarker levels in critically ill, ICU-admitted patients remain scarce.

Regarding ET-1 and HBP, we have previously shown an association between plasma levels of these biomarkers in experimental sepsis, where ET-receptor antagonism reduced plasma HBP levels, and ET-1 administration in non-septic conditions resulted in a dose-dependent increase in plasma HBP [48].

The aim of the study was to investigate the plasma levels of HBP and ET-1 at ICU admission in critical COVID-19 and their association with 60-day mortality and need for IMV.

## Methods

### Study design and participants

This observational cohort study was performed between April-October 2020 at the Karolinska University Hospital, Sweden, a tertiary referral centre, covering an urban area of approximately 2.4 million inhabitants. During this period, specific COVID-19 ICUs were established. Patients were admitted to these units primarily due to severe respiratory failure requiring advanced respiratory support, such as high-flow nasal oxygen, non-invasive mechanical ventilation, or IMV.

Patients 18 years or older with confirmed SARS-CoV-2 infection by polymerase chain reaction were eligible for inclusion upon admission to the COVID-ICU. The main reasons for not including all eligible patients were limited research nurse availability, failure to obtain informed consent from the patient or closest relative, or inability of the patient to understand the study protocol.

For comparison of HBP and ET-1 values, plasma samples were collected from ten ICU-admitted trauma patients.

### Data collection

Admittance data and clinical parameters were retrieved from the Swedish Intensive Care Register and electronic patient data management systems (Clinisoft^®^, GE, Barrington IL), including patient medical records (Take Care^®^, CompuGroup Medical, Koblenz, Germany). Information was collected on demographic data, laboratory results, comorbidities, hospital and ICU admissions, respiratory and circulatory parameters, and outcomes such as mortality and continuous renal replacement therapy (CRRT).

Data was collected during ICU stay, until discharge or death, whichever occurred first. Variables of interest included age, sex, body mass index (BMI), and comorbidities. Simplified acute physiology score (SAPS) III, time from symptom onset to hospital admission, ICU admission, and death, as well as laboratory data, were recorded. Treatments such as prone positioning, muscle relaxation, CRRT, steroids, and specific therapies such as interleukin (IL)-blockers and anticoagulation, were documented. Clinical outcome data were available for all patients.

### Blood sampling and biomarker analysis

Blood samples were drawn within 24 h of ICU admission from an indwelling cannula into plasma preparation tubes (PPT) (BD Vacutainer^®^PPT TM, Becton Dickinson, New Jersey, USA). The tubes were centrifuged in 1200 G for 10 min at 4 °C and stored at -80 °C. After storage the plasma was separated from the tubes and stored again at -80° until biomarker analysis.

Heparin-binding protein was analysed in duplicates using the Axis-Shield HBP microtiter plate enzyme-linked immunosorbent assay (ELISA, Axis-Shield Diagnostics, Dundee, United Kingdom). Endothelin-1 was analysed using the Quantikine® ELISA (R&D Systems® Inc., Minneapolis, USA). Routine laboratory measurements were obtained from the established clinical routine laboratory assays at the Karolinska University Hospital Laboratory.

### Outcomes

The primary outcome was the association between plasma levels of HBP and ET-1 in critical COVID-19 disease and 60-day mortality from hospital admission. The secondary outcome was the association between these levels and the need for subsequent IMV during the ICU stay.

### Statistics

Nonparametric methods were used to analyse data. Comparisons of continuous variables were performed using the Mann-Whitney two-tailed test. Categorical variables were assessed using Fisher´s exact test. A p-value of < 0.05 was considered statistically significant. Continuous data are presented as median ± interquartile range (IQR) and depicted as box plots. Categorical data are presented as proportions and percentages.

Outcomes were assessed using multivariable logistic regression analyses, adjusted for age, sex, and BMI. Goodness of fit and model discrimination were determined using the Hosmer-Lemeshow test. Comparison between plasma HBP and ET-1 collected in PPT tubes or citrated tubes was performed using Spearman correlation as well as Mann-Whitney two-tailed test.

The predictive properties of the biomarkers were analysed with receiver operating characteristic curves (ROC) and presented as area under the curve (AUC) with corresponding confidence interval (CI). Statistical analyses were performed using Prism 9 (Graphpad Inc., San Diego, USA).

## Results

A total of 96 patients with critical COVID-19 were included and analysed in the study, with the vast majority (96%) enrolled between April and June 2020. Heparin-binding protein measurements were available for 78 patients and ET-1 measurements for 92 patients.

In four patients HBP samples were missing, and in 14 individuals, complications with the Axis- Shield ELISA rendered the values unusable. Additionally, ET-1 samples were missing in four patients.

**Fig. 1:**
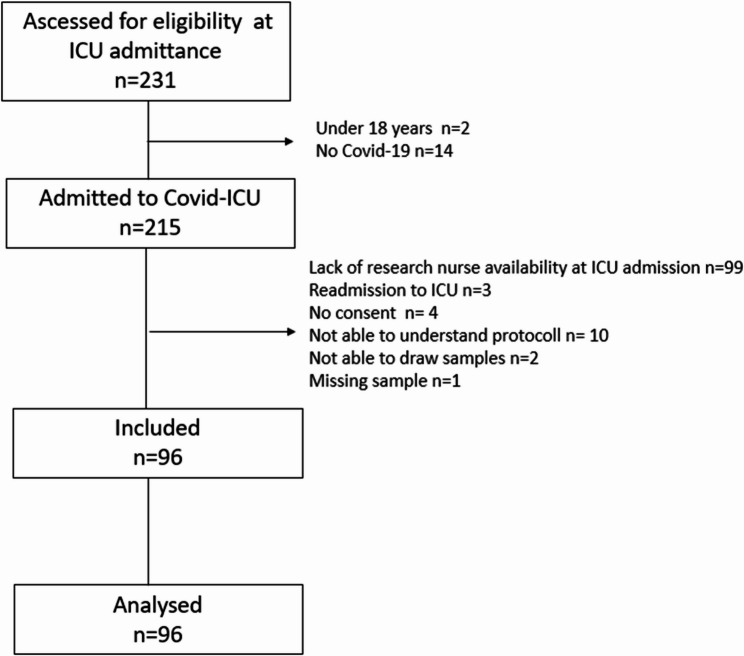
Patient flowchart. ICU, intensive care unit

A flowchart and demographic data for inclusion of critical COVID-19 patients admitted to ICU is presented in Fig. 1 and in Table 1.

**Table 1:** Patient characteristics and outcomes for 60-day survivors and non-survivors

Patient characteristics	All (*n* = 96)	Dead (*n* = 17)	Surviving (*n* = 79)	*P*
Age, years	61 (53–66)	65 (57–68)	60 (53–66)	ns
Male	73 (76)	17 (100)	56 (71)	**
BMI	28 (25–31)	26 (24–32)	28 (26–31)	ns
Comorbidity				
No previous medical history	23 (24)	2 (12)	21 (27)	ns
Cardiovascular disease	54 (56)	9 (53)	45 (57)	ns
Lung disease	20 (21)	4 (24)	16 (20)	ns
Diabetes I or II	31(32)	5 (29)	26 (33)	ns
Chronic kidney disease	7 (7)	2 (12)	5 (6)	ns
Chronic GI/liver disease	7(7)	1 (6)	6 (8)	ns
Immunocompromised	9 (9)	3 (24)	6 (9)	ns
Any malignancy	11 (11)	4 (24)	7 (9)	ns
Time from symptom to hospital admission, days	8 (6–11) *n* = 94	6 (4–12) *n* = 16	8 (6–11) *n* = 78	ns
Time from symptom to ICU admission, days	10 (8–14) *n* = 94	10 (6–13) *n* = 16	10 (8–14) *n* = 78	ns
Time from symptom to death, days		33 (23–45) *n* = 16		
Time from hospital admission to death, days		22 (18–38) *n* = 17		
SAPS III score	51 (47–57)	51 (49–62)	49 (46–56)	ns
Hospital duration, days	21 (16–38)	22 (19–38)	21 (15–39)	ns
ICU duration, days	12 (5–21)	22 (17–36)	9 (5–16)	****
CRRT	12 (13)	6 (35)	6 (8)	**
IMV	61 (64)	16 (94)	45 (57)	**
IMV days	9 (0–18)	17 (14–35)	6 (0–14)	****
Prone position	69 (72)	16 (94)	53 (67)	*
Steroids first 7 days at ICU	53 (55)	14 (82)	39 (49)	*
Steroids before HBP test	24 (25) *n* = 93	5 (29) *n* = 15	19 (24) *n* = 78	ns
Remdesivir	4 (4)	2 (12)	2 (3)	ns
Monoclonal antibody	21 (22)	4 (24)	17 (22)	ns
LMWH dose before HBP test	5000 (5000–7500) *n* = 91	5000 (5000–7500) *n* = 15	5000 (5000–7500) *n* = 76	ns
HBP ng/ml	150 (47–299) *n* = 78	193 (34–545) *n* = 15	141 (50–292) *n* = 63	ns
ET-1 pg/ml	1.6 (1.2–1.9) *n* = 92	1.7 (1.4–1.9) *n* = 16	1.6 (1.1–1.9) *n* = 76	ns
WBC x10(9)/L	8.4 (6.4–10.9)	9.1 (5.8–11.7)	8.2 (6.5–10.7)	ns
PCT µg/L	0.6 (0.2–1.4)	0.9 (0.5–1.7)	0.5 (0.2–1.1)	ns
CRP mg/L	176 (123–257)	207 (126–261)	169 (114–255)	ns
HBP/WBC ratio	16.2 (6.7–41.0) *n* = 78	24.2 (6.3–67.3) *n* = 15	14.6 (6.8–39.0) *n* = 63	ns
D-Dimer mg/L	1.2 (0.7–2.6) *n* = 74	1.3 (0.8–3.8) *n* = 13	1.2 (0.7–2.6) *n* = 61	ns
Thrombocytes x10(9)/L	265 (202–312)	240 (217–297)	270 (199–330)	ns
IL-6 ng/L	132 (62–324) *n* = 46	215 (129–393) *n* = 9	110 (52–308) *n* = 37	ns
Ferritin µg/L	1419 (699–2153) *n* = 51	1738 (1272–2052) *n* = 10	1251 (607–2183) *n* = 41	ns

Data displayed as median (interquartile range) for continuous variables and number (%) for categorical variables

*BMI* body mass index, *GI* gastrointestinal, *ICU* intensive care unit, *SAPS* simplified acute physiology score, *CRRT* continuous renal replacement therapy, *IMV* invasive mechanical ventilator, *HBP* heparin-binding protein, *LMWH* low molecular weight heparin (value in units), *ET-1* endothelin-1, *WBC* white blood cell, *PCT* procalcitonin, *CRP* C-reactive protein, *IL-6* interleukin-6

Monoclonal antibody refers to IL-1 or IL-6 blockers. Groups compared with Mann-Whitney two tailed test for continuous data and Fisher exact test for categorical data. * Denotes *p* < 0.05, ** *p* < 0.01, **** denotes *p* < 0.0001

### Patient characteristics, treatment and blood sampling

Patient characteristics and outcomes are summarized in Tables 1 and 2. The median time from the onset of COVID-19 symptoms to hospital admission was 8 days (IQR 6–11), and to ICU admittance, 10 days (IQR 8–14).

**Table 2:** Patient characteristics and outcome of patients treated with or without invasive mechanical ventilation

Patient characteristics	All (*n* = 96)	IMV YES (*n* = 61)	IMV NO (*n* = 35)	*P*
Age	61 (53–66)	62 (57–67)	57 (50–65)	*
Male	73 (76)	45 (73)	28 (80)	ns
BMI	28 (25–31)	28 (25–31)	28 (25–31)	ns
Comorbidity				
No previous medical history	23 (24)	14 (23)	9 (26)	ns
Cardiovascular disease	54 (56)	34 (56)	20 (57)	ns
Lung disease	20 (21)	13 (21)	7 (20)	ns
Diabetes I or II	31(32)	18 (30)	13 (37)	ns
Chronic kidney disease	7 (7)	6 (10)	1 (3)	ns
Chronic GI/liver disease	7(7)	7 (11)	0 (0)	*
Immunocompromised	9 (9)	7 (12)	2 (6)	ns
Any malignancy	11 (11)	9 (15)	2 (6)	ns
Time from symptom to hospital admission, days	8 (6–11) *n* = 94	8 (5–11)	10 (7–12)	ns
Time from symptom to ICU admission, days	10 (8–14) *n* = 94	10 (7–13)	11 (9–14)	ns
Dead	17 (18)	16 (26)	1 (3)†	**
Time from symptom to death, days		35 (24–45) *n* = 15	5	
Time from hospital admission to death, days		25 (18–38) *n* = 16	1	
SAPS	51 (47–57)	53 (49–60)	47 (44–50)	****
Hospital duration, days	21 (16–38)	30 (21–49)	14 (10–17)	****
ICU duration days	12 (5–21)	17 (12–27)	4 (2–6)	****
CRRT	12 (13)	12 (20)	0 (0)	**
IMV days		15 (9–27)		
IMV on ICU admission day		31 (51)		
IMV during the first 3 ICU days		58 (95)		
Prone position	69 (72)	42 (69)	27 (77)	ns
Steroids first 7 days at ICU	53 (55)	32 (52)	14 (40)	ns
Steroids before HBP test	24 (25) *n* = 93	12(20)	12 (34)	ns
Remdesivir	4 (4)	4 (7)	0 (0)	ns
Monoclonal antibody	21 (22)	14 (23)	7 (20)	ns
LMWH dose before HBP test	5000 (5000–7500) *n* = 91	5000 (5000–7500) *n* = 56	5000 (5000–7500) *n* = 35	ns
HBP ng/ml	150 (47–299) *n* = 78	150 (40–430) *n* = 48	160 (59–284) *n* = 30	ns
ET-1 pg/ml	1.6 (1.2–1.9) *n* = 92	1.7 (1.3-2.0) *n* = 57	1.4 (1.0-1.7) *n* = 35	**
WBC x10(9)/L	8.4 (6.4–10.9)	8.2 (6.1–11.2)	8.5 (6.7–10.0)	ns
PCT µg/L	0.6 (0.2–1.4)	0.9 (0.4–1.8)	0.3 (0.1–0.6)	****
CRP mg/L	176 (123–257)	207 (136–284)	133 (99–235)	**
HBP/WBC ratio	16.2 (6.7–41.0) *n* = 78	18.0 (5.6–49.0) *n* = 48	15.0 (7.3–35.3) *n* = 30	ns
D-Dimer mg/L	1.2 (0.7–2.6) *n* = 74	1.3 (0.8–2.8) *n* = 45	1.1 (0.6–1.8) *n* = 29	ns
Thrombocytes x10(9)/L	265 (202–312)	262 (203–305)	277 (200–363)	ns
IL-6 ng/L	132 (62.3–324.0) *n* = 46	211 (80-454.8) *n* = 30	74 (31.3-221.8) *n* = 16	*
Ferritin µg/L	1419 (699–2153) *n* = 51	1419 (824–2209) *n* = 33	1285 (537–2168) *n* = 18	ns

Data displayed as median (interquartile range) for continuous variables and number (%) for categorical variables

*BMI* body mass index, *GI* gastrointestinal, *ICU* intensive care unit, *SAPS* simplified acute physiology score, *CRRT* continuous renal replacement therapy, *IMV* invasive mechanical ventilator, *HBP* heparin-binding protein, *LMWH* low molecular weight heparin (value in units), *ET-1* endothelin-1, *WBC* white blood cell, *PCT* procalcitonin, *CRP* C-reactive protein, *IL-6* interleukin-6

Monoclonal antibody refers to IL-1 or IL-6 blockers. Groups compared with Mann-Whitney two tailed test for continuous data and Fisher exact test for categorical data. *Denotes *p* < 0.05, ** *p* < 0.01, **** denotes *p* < 0.0001. † One patient died with restrictions of medical treatments, including mechanical ventilation

The cohort was characterised by severe respiratory failure, with 61 of 96 patients (64%) requiring IMV during their ICU stay. The vast majority (76%) were male and 76% had pre-existing medical conditions. The median length of ICU stay was 12 days (IQR 5–21).

All patients exhibited laboratory signs of inflammation, including elevated C-reactive protein (CRP), procalcitonin (PCT), interleukin-6 (IL-6) and ferritin levels, while white blood cell count (WBC) remained within normal ranges.

The median time from ICU admission to HBP and ET-1 blood sampling was 15 h (IQR 9–23). In most patients (72%), other laboratory data measurements were taken within six hours before or after the HBP and ET-1 sample. For the remaining 28%, samples were taken within 12 h of the HBP/ET-1 sample.

No patient was treated with hydroxychloroquine, while four patients received remdesivir. Approximately half of the patients received steroids during the first seven ICU days and 25% of all patients had been given steroids before blood sampling. Similarly, 22% of the patients received monoclonal antibodies against IL-1 or IL-6 of which seven patients received this treatment before blood sampling.

A comparison between patients receiving steroids and monoclonal antibodies showed no significant influence on HBP or ET-1 levels (data not shown).

All patients, except one, received low molecular weight heparin (LMWH), specifically dalteparin, during their ICU stay. The exception was a patient treated with a heparin infusion due to extracorporeal membrane oxygenation, which was initiated after blood sampling.

### Comparison of test tubes

**Fig. 2:**
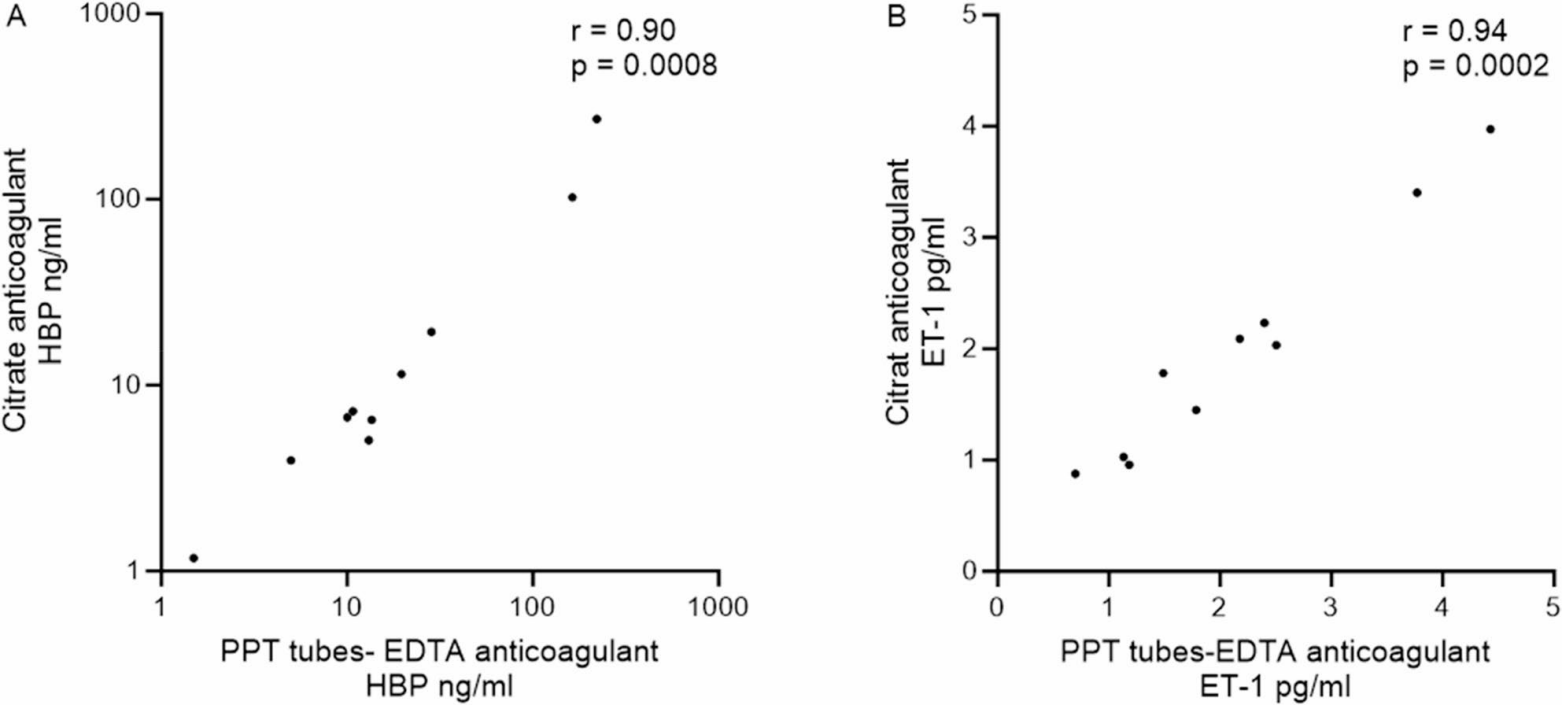
Comparison of heparin-binding protein (HBP) and endothelin-1 (ET-1) measurements in samples collected in plasma preparation tubes (PPT) containing EDTA and citrated test tubes. Correlation between HBP measurements (**A**) and ET-1 measurements (**B**) PPT tubes or citrated test tubes

To investigate if PPT test tubes (containing EDTA as anticoagulant and a gel plug separating plasma during centrifugation) interfere with the HBP-ELISA which is recommended for human citrated plasma only, we compared PPT tubes with standard tubes containing citrate. In the ten severely injured trauma ICU patients the HBP and ET-1 levels using the PPT tubes were 13.3 ng/ml (IQR 8.8–62.1) and 2.0 pg/ml (IQR 1.2–2.8), respectively. The levels in citrated tubes were 7.0 ng/ml (IQR 4.8–40.1) and 1.9 pg/ml (IQR 1.0-2.5), respectively. The HBP and ET-1 levels in the PPT test tubes were consequently higher than in the citrate tubes but the difference was not significant (Supplementary Table 1). HBP and ET-1 values correlated strongly between tube types (Fig. 2).

## Heparin-binding protein, endothelin-1 and patient outcomes

### Mortality

The 60-day mortality after hospital admission was 17.8%. All the deceased patients were male and died during hospital stay. The median time from COVID-19 symptoms to death was 33 days (IQR 23–45).

**Fig. 3:**
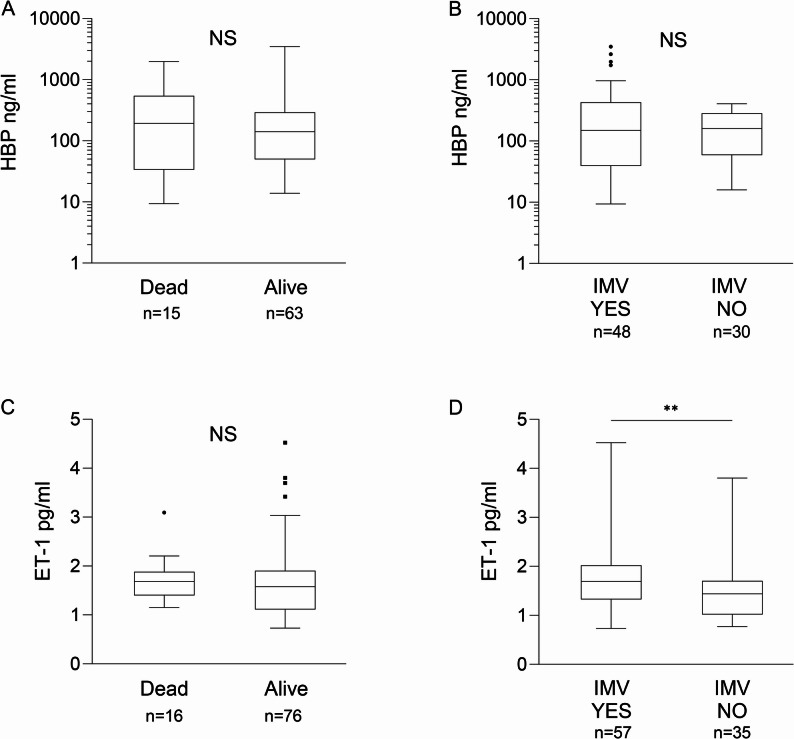
Heparin-binding protein (HBP) and endothelin-1 (ET-1) levels in critical COVID-19 patients. 60-day survivors vs. non-survivors (**A** and **C**, respectively). Patients requiring invasive mechanical ventilation (IMV) or not (**B** and **D**, respectively). Data displayed as Tukey box and whisker plots with the median indicated by the horizonal line. NS denotes a non-significant difference (*p* ≥ 0.05). ** denotes *p* < 0.01

The median HBP and ET-1 levels at admission were 150 ng/ml (IQR 47–299) and 1.6 pg/ml (IQR 1.2–1.9), respectively. There was no significant difference in plasma HBP levels between patients who survived and those who died (141 ng/ml; IQR 50–292 and 193 ng/ml; IQR 34–545, respectively), *p* = 0.64, (Table 1; Fig. 3A). Similarly, there was no difference in plasma ET-1 levels between surviving patients (1.6 pg/ml; IQR 1.1–1.9) and deceased patients (1.7 pg/ml; IQR 1.4–1.9), *p* = 0.30, (Table 1; Fig. 3C).

Patients who died were more frequently treated with IMV as well as CRRT. They also had a longer ICU stay compared to surviving patients (Table 1). There was no significant difference in CRP, WBC, ferritin, D-dimer, or thrombocyte count between patients who died and those who survived. Heparin-binding protein or ET-1 levels did not correlate with any of these laboratory parameters (data not shown), except that HBP correlated with WBC, (*r* = 0.33, *P* = 0.004). Moreover, there was no significant correlation between HBP and ET-1, (Supplementary Fig. 1).

In a logistic regression model adjusted for age, sex and BMI, neither HBP nor ET-1 was significantly associated with mortality (OR 1.000; 95%CI 0.999–1.001 and OR 1.095; 95%CI 0.489–2.252, respectively). The model demonstrated good calibration using the Hosmer-Lemeshow test.

**Fig. 4:**
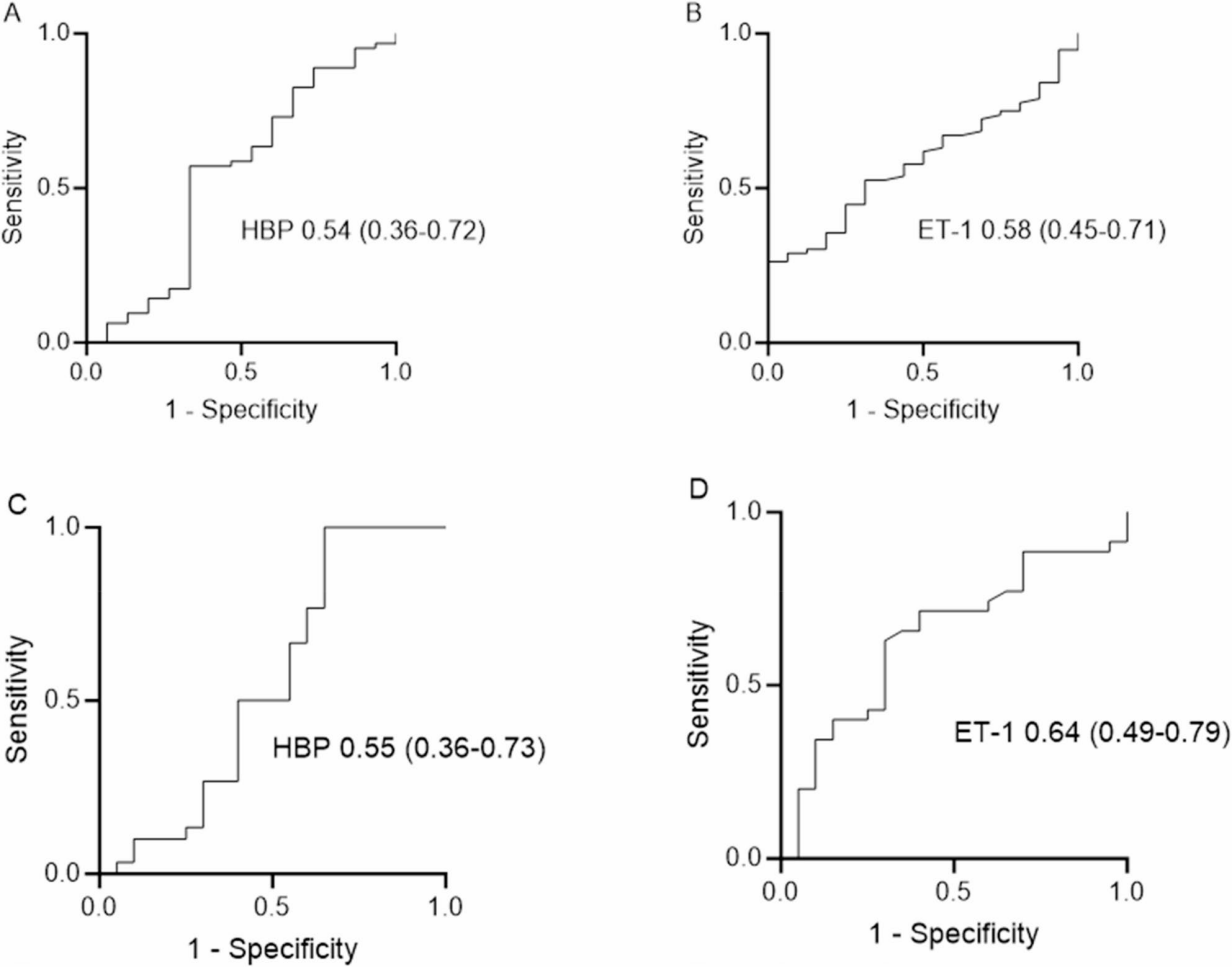
Predictive performance of biomarkers. Receiver operating characteristic (ROC) for prediction of mortality and invasive mechanical ventilation. **A** Heparin-binding protein (HBP) and 60-day mortality (*n* = 78), **B** Endothelin-1 (ET-1) and 60-day mortality (*n* = 92), **C** HBP and invasive mechanical ventilation (*n* = 50), **D** ET-1 and invasive mechanical ventilation (*n* = 55). Area under the curve (AUC) values and 95% confidence interval

Receiver operating characteristic curves were used to evaluate prediction of mortality in all patients. The AUC was non-significant for HBP, ET-1, CRP, PCT, WBC, HBP/WBC, thrombocyte count, D-dimer, IL-6 and ferritin (Fig. 4A and B; Supplementary Fig. 2). There was no difference in LMWH dosage before HBP sampling or during ICU stay between surviving patients and those who succumbed to the disease (Table 1).

### Mechanical ventilation

The majority of patients admitted to the ICU (61 out of 96; 64%) received IMV during their stay, with 58 (95%) of these patients being intubated within 72 h. One patient was intentionally managed solely with non-invasive ventilation due to end-stage lung disease.

The HBP levels upon admission were comparable between patients who required IMV and those who did not during their ICU stay (Table 2; Fig. 3B). However, ET -1 levels were significantly higher (1.7 pg/ml; IQR 1.3-2.0) in patients requiring IMV compared to those who did not (1.4 pg/ml; IQR 1.0-1.7), *p* = 0.004, (Table 2; Fig. 3D).

In a logistic regression model, adjusted for age, sex and BMI neither HBP nor ET-1 showed a significant association with IMV (OR 1.001; 95%CI 1.000-1.004 and OR 1.854; 95%CI 0.916–4.303, respectively). The model demonstrated good calibration.

Receiver operating characteristic curves were used to evaluate prediction of subsequent IMV after the initial plasma sampling. The AUCs were non-significant for both HBP and ET-1 (Fig. 4C and D).

Patients requiring IMV were significantly older. 20% of these patients received CRRT compared to none in non-IMV group. Similarly, they had significantly higher CRP levels with a median of 207 mg/L (IQR 136–284) compared to 133 mg/L (IQR 99–235) in the group that did not receive IMV. Procalcitonin and IL-6 at admission were also significantly higher, whereas other laboratory parameters were similar (Table 2). The majority (74%) were empirically treated with antibiotics during the first seven ICU days; however, no data were available on the actual rate of secondary bacterial pneumonia.

## Discussion

In this study, we measured plasma levels of HBP and ET-1 at ICU admission in critically ill COVID-19 patients. The HBP levels were markedly elevated, more than tenfold higher compared to a group of post-trauma ICU patients. There was no significant difference in plasma levels of HBP or ET-1 between 60-day survivors and non-survivors.

Heparin-binding protein levels were not associated with the need for IMV treatment whereas plasma ET-1 levels were higher in patients requiring IMV. However, after adjusting for sex, age and BMI, no significant association was observed between plasma ET-1 levels and need for IMV.

There are some previous studies in which HBP levels above 30 ng/ml in COVID-19 patients distinguished those who developed organ failure or severe respiratory failure requiring IMV from those with a more favourable disease course [24, 25]. Other studies have found increased mortality using a HBP cutoff at 13.5 or 35 ng/ml [49, 50]. In these studies, the patients were included already at hospital admission, a much earlier stage of the disease. Our study cohort had considerably higher HBP levels, with a median level of 150 ng/ml (IQR 47–299) at inclusion. A possible explanation is that all patients in our study had already developed signs of severe organ failure ten days after onset of a symptomatic virus infection. Furthermore, our patients exhibited remarkably high inflammatory markers. It appears that the discriminative properties of HBP are lost at these high levels. The lack of prognostic value of HBP in our cohort is likely explained by the stage of disease at which patients were sampled. Studies reporting prognostic value for HBP in COVID-19 enrolled patients earlier, at hospital rather than ICU admission and used lower discriminatory cutoffs (13.5–35 ng/ml) [24, 49, 50]. In contrast, all patients in our cohort had already progressed to critical illness requiring ICU admission, with a median HBP of 150 ng/ml, far exceeding these thresholds. At this advanced stage, HBP levels may be uniformly and markedly elevated across the entire cohort regardless of subsequent outcome, thereby losing their discriminatory capacity. This interpretation is supported by findings in critically ill H1N1 patients, where similarly elevated ICU admission HBP levels were not associated with ARDS or severe septic shock. These observations collectively suggest that HBP is most informative as a prognostic tool when measured earlier in the disease course, before a clinical ceiling effect is reached.

Interpreting HBP levels across studies should be approached with caution, as the timing of HBP sampling in relation to disease severity and progression may vary significantly. To our knowledge, no published data are available on HBP levels from the severe acute respiratory syndrome (SARS) outbreak in 2002–2004, a disease caused by SARS-CoV-1. However, in a study from Finland, IMV-treated patients with viral pneumonia caused by H1N1 (swine flu) had a median HBP level of 152 ng/ml at ICU admission, which aligns with the levels observed in our cohort [23]. Although the authors reported higher HBP levels in IMV-treated patients than in non-IMV-treated patients, they found no association between admission HBP levels and the development of ARDS or severe sepsis/septic shock. These findings, together with those of the current study, suggest that in certain critical forms of viral infections, HBP levels may be markedly elevated, but their discriminatory capacity is diminished.

According to the manufacturer of the HBP-Axis-Shield ELISA, sampling should be done in test tubes using citrate as an anticoagulant. To ensure that the high levels of HBP were not caused by the PPT/EDTA sampling tubes, we aimed to compare HBP measurements between PPT/EDTA and citrate tubes. As the number of COVID-19 patients decreased by the time the HBP measurements were available, we could not include COVID-19 patients for this comparison. Therefore, we included ten post-trauma ICU patients with presumably elevated levels of HBP for these measurements. Although, these patients had lower HBP levels (13.3 ng/ml; IQR 8.8–62.1), we found a strong correlation between HBP levels analysed from different tubes. It should be noted that the tube comparison was performed in only ten trauma ICU patients, which limits the statistical power to detect a potential systematic bias between tube types. Although the correlation was strong and the difference in absolute values was not statistically significant, a measurement bias cannot be excluded with certainty. This should be considered when comparing our HBP values directly to those reported in studies using citrate tubes. However, given the markedly elevated HBP levels observed in our cohort, even a moderate overestimation related to tube type would be unlikely to account for the magnitude of elevation or to substantially alter the main conclusions. Post-trauma ICU patients were selected as comparators because banked samples collected under identical conditions were available from an ongoing study at our institution, and because trauma patients are known to have elevated HBP levels [51], making them suitable for the tube-type validation at clinically relevant biomarker concentrations. However, this group is not an ideal biological reference population for COVID-19. A healthy control group would have provided a clearer reference range, and non-COVID ARDS patients would have allowed more disease-specific comparisons. The absence of such control groups is acknowledged as a limitation of the present study.

As the name implies, heparins bind to HBP, and our group, along with others, has previously shown that heparins, including LMWH, can elevate the plasma HBP levels [52, 53]. Therefore, it is possible that LMWH contributed to the high levels found in our study, as the majority of patients received dalteparin before blood sampling. Considering that several hours had passed between LMWH administration in most patients, the effects of LMWH on plasma HBP concentration might have diminished [53]. Unfortunately, anti-factor Xa levels were not routinely measured.

There are a few studies reporting repeated measures of HBP during COVID-19 disease. The studies show varying levels depending on when the sample is taken during the course of the disease, but generally, decreasing levels are observed once clinical stability is reached [24–26, 54]. It was not possible to measure HBP levels repeatedly in our study.

In a recently published study HBP levels were repeatedly measured during ICU stay in critical COVID-19 patients. Notably, all patients were treated with therapeutic doses of LMWH or heparin infusion, and the overall mean HBP level was 202 ng/ml [54]. Some patients had extremely high values, similar to those observed in our cohort. Moreover, HBP levels were strongly associated with disease severity and remained elevated throughout the ICU stay. Interestingly, patients receiving heparin had higher plasma HBP compared to those treated with LMWH. However, the effects and timing of heparin administration in relation to blood sampling was not considered [54].

The pathophysiology of respiratory dysfunction in ARDS and especially when caused by SARS-CoV-2 infection is not fully understood. Endothelial and epithelial dysfunction, along with systemic inflammation and microvascular hyperpermeability, are key features of ARDS, regardless of the underlying cause [55]. It is highly probable that HBP is involved in this process, as both in vitro and in vivo studies have demonstrated its ability to open the endothelial lining [12, 29, 56].

Endothelin-1 contributes to the pathophysiology of pulmonary artery hypertension, commonly seen in ARDS, and is associated with proliferative, fibrotic, and thrombogenic vascular remodelling [36, 57]. We found slightly elevated levels of ET-1 in our patients compared to the normal levels reported in healthy subjects [43, 44] and those provided from the manufacturer of our ELISA. Patients receiving IMV had higher levels compared to patients not receiving this treatment. As the majority of the patients required IMV treatment shortly after ICU admission, we speculate that this group may have been more hypoxic at the time of blood sampling given that hypoxia is known to increase ET-1 secretion [58, 59]. In addition, the clearance of ET-1 could be disrupted in ARDS caused by COVID-19. Animal and human studies have established the pulmonary vascular endothelium as a major site for ET-1 removal through the endothelial ET_B_ subtype receptors [60–64]. Therefore, the elevated levels of ET-1 could be caused by increased production, reduced removal or a combination of both. Patients requiring IMV were also significantly older, which could have impacted the ET-1 levels [65–67]. There are a few studies confirming high levels of ET-1 in COVID-19, with increasing levels in severely ill compared to patients with milder disease [30]. In addition, the levels seem to remain elevated even three months after the infection [43].

In a previous study on porcine endotoxemia, we observed an association between HBP and ET-1, where systemic ET-receptor antagonism markedly reduced plasma HBP levels, while ET-1 administration in control animals dose-dependently increased plasma HBP levels [48]. However, in the present study on a severe viral disease, we found no correlation between plasma HBP and ET-1. Endothelial dysfunction is a complex phenomenon, and other mechanism may also be involved.

This study has several limitations. It was conducted exclusively during the first wave of the COVID-19 pandemic in 2020, a period characterised by limited clinical experience and rapidly evolving treatment practices. Decisions regarding timing of IMV initiation, corticosteroid use, and immunotherapy were made under considerable uncertainty, and protocols were not yet standardised. Patient selection was furthermore constrained by research nurse availability and consent procedures, which may have introduced selection bias. The clinical phenotype of COVID-19, the circulating variants, and the immune status of the population have changed substantially since 2020, and it therefore remains uncertain whether our findings are generalisable to later pandemic waves or to disease in vaccinated individuals. It is a single-centre study with a limited cohort of COVID-19 patients. Moreover, as we could only obtain samples at admission, it was not possible to assess temporal changes in HBP in relation to outcome measures. The restriction to a single time-point measurement at ICU admission is a significant limitation. Inflammatory and endothelial biomarkers such as HBP and ET-1 are dynamic and may evolve substantially over the course of critical illness. A single admission value captures only a snapshot of this process and may not reflect subsequent trajectories. Serial measurements might have provided greater discriminatory power. Patients with persistently elevated or rising HBP could represent a higher-risk subgroup compared to those with declining levels, a distinction that a single admission sample cannot capture. Future studies should incorporate repeated biomarker sampling to assess temporal dynamics and their relationship to clinical outcomes. The study lacks HBP and ET-1 samples from healthy controls and includes only a small group of post-trauma ICU patients. Nevertheless, we believe that that the HBP levels observed in our cohort are among the highest reported in the literature and significantly exceed those described in other severe conditions, such as sepsis [22], cardiac arrest [68], cardiac surgery [52], or trauma [51]. Patients were included at a time when COVID-19 was a novel disease, and clinical experience regarding treatment strategies, such as timing of IMV initiation, was limited. Similarly, uncertainties regarding the timing and dosing of steroids, immunotherapy, and anticoagulative treatments may have influenced outcomes. As this study was conducted during the first wave of the COVID-19 pandemic, it remains uncertain whether our findings are applicable to later variants of the virus or to disease presentation in vaccinated individuals.

## Conclusions

We found markedly elevated plasma levels of HBP at ICU admission in patients with critical COVID-19. Among these patients, no association was found between plasma HBP and 60-day mortality or the need for IMV during the ICU stay. Plasma levels of ET-1 were modestly elevated upon ICU admission but were not associated with mortality or the need for IMV. These findings should be interpreted in the context of the study’s limitations: the single-centre design and relatively small cohort size limit generalisability, biomarker measurements were obtained at a single time point only, and the study lacks a well-matched healthy control group. Future multicentre studies with serial biomarker measurements are needed to confirm and extend these findings.

## Supplementary Information

Supplementary Material 1.

## Data Availability

The datasets used and/or analysed during the current study are available from the corresponding author on reasonable request.

## Abbreviations

AUC: Area under the curve
ARDS: Acute respiratory distress syndrome
BMI: Body mass index
CI: Confidence interval
CRP: C-reactive protein
CRRT: Continuous renal replacement therapy
ELISA: Enzyme-linked immunosorbent assay
ET-1: Endothelin-1
HBP: Heparin-binding protein
ICU: Intensive care unit
IQR: Interquartile range
IL: Interleukin
IMV: Invasive mechanical ventilation
LMWH: Low molecular weight heparin
OR: Odds ratio
PCT: Procalcitonin
PPT: Plasma preparation tubes
PMN: Polymorphonuclear neutrophils
ROC: Receiver operating characteristic curve.
SAPS: Simplified acute physiology score
SARS: Severe acute respiratory syndrome
WBC: White blood cell count
