# Supplementary material for: Heparin-binding protein and Endothelin-1 in critical COVID-19
Source: BMC Anesthesiol. 2026 Jun 23;26:379. doi: 10.1186/s12871-026-04050-0 (PMC13292514; doi:10.1186/s12871-026-04050-0)
Supplement: Supplementary file 1 — Supplementary Material 1. [file 12871_2026_4050_MOESM1_ESM.docx]

**Supplement**

**Comparison of test tubes on HBP and ET-1 measurements**

The Axis-Shield ELISA for HBP analysis is recommended for human citrated plasma only. The samples from COVID-19 patients were collected in PPT test tubes containing ethylenediaminetetraacetic acid (EDTA) as an anticoagulant and a gel plug to separate plasma during centrifugation. The Quantikine^®^ ELISA for ET-1 can be used with both EDTA- and citrate-treated plasma. Therefore, we investigated ten ICU admitted trauma patients, comparing PPT test tubes with citrated test tubes to measure both HBP and ET-1.

S-Table 1. Characteristics and comparison of HBP and ET-1 levels measured in PPT and citrate tubes in ten ICU-admitted trauma patients.

| **ICU admitted traumapatients n=10** | **HBP and ET-1**  **PPT tubes** | **HBP and ET-1 citrate tubes** | **P value** |
| --- | --- | --- | --- |
| ISS score | 24.5 (21.8-30.3) |  |  |
| HBP ng/ml | 13.3 (8.8-62.1) | 7.0 (4.8-40.1) | 0.35 |
| ET-1 pg/ml | 2.0 (1.2-2.8) | 1.9 (1.0-2.5) | 0.68 |
| Age | 55 (38-70) |  |  |
| Male | 7 (70) |  |  |
| Time from hospital admission to blood sampling ( n=9) | 07:26  (0:13-24:25) |  |  |
| Death within 7 days | 2 (20) |  |  |
| IMV at day 1,2 or 3 post trauma | 10 (100) |  |  |
| IMV at 7 days post trauma | 3 (30) |  |  |

Data displayed as median (interquartile range) for continuous variables and number (%) for categorical variables except for time which is shown in median (minimum-maximum time i hours:minutes). HBP, heparin-binding protein; ET-1, endothelin-1; PPT, plasma preparation tubes; ICU, intensive care unit; ISS, Injury severity score; IMV, invasive mechanical ventilation.


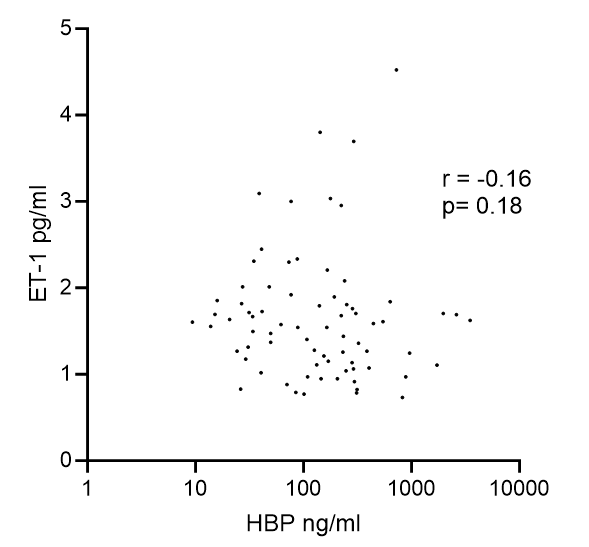


S-Fig 1. Correlation between heparin-binding protein (HBP) and endothelin-1 (ET-1). Levels were compared using Spearman correlation (n=74).

S-Fig 2. Predictive performance of biomarkers. Receiver operating characteristic (ROC) for prediction of 60-day mortality. A) C-reactive protein (CRP, n=96), B) Procalcitonin (PCT, n=96), C) White blood cells (WBC, n=96), D) HBP/WBC (heparin-binding protein/WBC, n=78) E) Thrombocytes (n=96), F) D-dimer (n=74), G) Interleukin-6 (IL-6, n=46), H) Ferritin (n= 51). Area under the curve (AUC) values and 95% confidence interval.

| **ICU admitted traumapatients n=10** | **HBP and ET-1**  **PPT tubes** | **HBP and ET-1 citrate tubes** | **P value** |
| --- | --- | --- | --- |
| ISS score | 24.5 (21.8-30.3) |  |  |
| HBP ng/ml | 13.3 (8.8-62.1) | 7.0 (4.8-40.1) | 0.35 |
| ET-1 pg/ml | 2.0 (1.2-2.8) | 1.9 (1.0-2.5) | 0.68 |
| Age | 55 (38-70) |  |  |
| Male | 7 (70) |  |  |
| Time from hospital admission to blood sampling ( n=9) | 07:26  (0:13-24:25) |  |  |
| Death within 7 days | 2 (20) |  |  |
| IMV at day 1,2 or 3 post trauma | 10 (100) |  |  |
| IMV at 7 days post trauma | 3 (30) |  |  |

S-Table 1. Characteristics and comparison of HBP and ET-1 levels measured in PPT and citrate tubes in ten ICU-admitted trauma patients. Data displayed as median (interquartile range) for continuous variables and number (%) for categorical variables except for time which is shown in median (minimum-maximum time i hours:minutes). HBP, heparin-binding protein; ET-1, endothelin-1; PPT, plasma preparation tubes; ICU, intensive care unit; ISS, Injury severity score; IMV, invasive mechanical ventilation.
